# Analytical Description of Degradation-Relaxation Transformations in Nanoinhomogeneous Spinel Ceramics

**DOI:** 10.1186/s11671-016-1722-0

**Published:** 2016-11-14

**Authors:** O. Shpotyuk, M. Brunner, I. Hadzaman, V. Balitska, H. Klym

**Affiliations:** 1 Jan Dlugosz University in Czestochowa, 13/15, Armii Krajowej str., 42200 Czestochowa, Poland; 2 Vlokh Institute of Physical Optics, 23, Dragomanov str., Lviv, 79005 Ukraine; 3 Technische Hochschule Köln/University of Technology, Arts, Sciences, 2, Betzdorfer Strasse, Köln, 50679 Germany; 4 Drohobych Ivan Franko State Pedagogical University, 24, I. Franko str., Drohobych, 82100 Ukraine; 5 Lviv State University of Life Safety, 35, Kleparivska str., Lviv, 79007 Ukraine; 6 Lviv Polytechnic National University, 12, Bandera str., Lviv, 79013 Ukraine

**Keywords:** Nanoinhomogeneity, Relaxation, Stretched-exponential, Compressed exponential, Kinetics, Spinel

## Abstract

Mathematical models of degradation-relaxation kinetics are considered for jammed thick-film systems composed of screen-printed spinel Cu_0.1_Ni_0.1_Co_1.6_Mn_1.2_O_4_ and conductive Ag or Ag-Pd alloys. Structurally intrinsic nanoinhomogeneous ceramics due to Ag and Ag-Pd diffusing agents embedded in a spinel phase environment are shown to define governing kinetics of thermally induced degradation under 170 °C obeying an obvious non-exponential behavior in a negative relative resistance drift. The characteristic stretched-to-compressed exponential crossover is detected for degradation-relaxation kinetics in thick-film systems with conductive contacts made of Ag-Pd and Ag alloys. Under essential migration of a conductive phase, Ag penetrates thick-film spinel ceramics via a considerable two-step diffusing process.

## Background

Disordered solids prepared by rapid cooling or quenching from a high-temperature fluid (liquid, melt) state compose a rich class of practically important materials known as *jammed systems* ranging from foams to soft glasses, dense colloidal gels, concentrated emulsions, clay suspensions, polymer nanocomposites, nanoparticle-filled supercooled liquids, glassy polymer melts, polymer nanocomposites disturbed by internal stress, and even some kinds of metallic glasses, etc. [1–7]. These materials realize their functionality due to specific structural inhomogeneities frozen at atomic and/or sub-atomic length scales, thus significantly modifying their physical-chemical properties, as well as materials’ response to different degradation-relaxation inputs. With tending towards equilibrium in the controlled parameter *N*(*t*), such systems obey compressed exponential relaxation (CER) kinetics (e.g., super-exponential), which is faster than simple exponential decay:

1 $$ {N}_{\eta }(t)= \exp \left[{\left(-\frac{t}{\tau}\right)}^{\beta}\right] $$

with scaling (compressing) exponent *β* > 1 and characteristic decay time *τ* varied (in dependence on scattering vector *q*) as *q* ^−1^, instead of *q* ^−2^ proper to conventional thermally activated diffusion [1, 2]. As to microscopic nature, these features can be well explained in terms of ultraslow ballistic motion of intrinsic scatters under internal stress frozen at a so-called *jamming transition* in structurally inhomogeneous systems like colloidal gels [1]. Built-in stress fields can be developed in these materials around some structural entities (such as embedded nanoparticles) to counter reduction in an entropy due to interaction with the surrounding. Therefore, the *β* value in Eq. (1) can serve as a quantitative measure of internal stress developed in such jammed systems under their quenching towards a metastable state. It is noteworthy that many of such systems being in a more uniform but still inhomogeneous state (due to structural evolution under changed chemistry or preparation conditions technology) demonstrate non-exponential kinetics mostly defined as stretched-exponential relaxation (SER), which is slower than simple single-exponential (e.g., sub-exponential) in full accordance the same with Eq. (1), but with non-negative below-unity stretching index (0 < *β* < 1) [8, 9]. Structural discrepancies between ingredients composing such systems play a decisive role defining *stretched-to-compressed non-exponential crossover* in the degradation-relaxation kinetics.

One of the typical examples showing crossover in non-exponential relaxation kinetics concerns diffusion problems, particularly protein folding [10–13] such as those occurring in photo-switchable α-helix due to *cis-trans* transition in polymer chains arrangement [14]. In equilibrium or a small-perturbation regime, these polymer materials have a strong tendency to yield SER kinetics (0 < *β* < 1). But under some conditions allowing perturbation from equilibrium (due to appearance of a so-called *downhill driving force* in an overall free energy landscape of a system), the measurements performed in direction to approach most sensitive employed experimental observables reveal CER kinetics (sometimes also termed as *squeezed-exponential*) with *β* > 1 rather than SER [12].

In this work, we shall try to track specificity of this stretched-to-compressed non-exponential crossover in thermally induced electric degradation-relaxation phenomena revealed in solid systems composed of the same heterogeneous matrix with different types of diffusion-able agents (diffusants), these being exemplified by thick-film structures of spinel transition-metal manganites (Cu, Ni, Co, Mn)_3_O_4_ with screen-printed metallic conductive layers formed of “pure” Ag or Ag-based pastes. Previously, some of the current authors disclosed that thick-film structures with Ag contacts, being subjected to thermal storage at elevated 170 °C temperature during a few hundreds of hours, demonstrated negative relative resistance drift (RRD) of 5–6% obeying CER kinetics with compressing exponent *β* = 1.6 [15, 16]. The negative RRD is also expected for thick films with screen-printed electrodes based on some Ag alloys (such as Ag-Pd conductive paste C1216), but governing relaxation kinetics seems to be rather changed instead [17–19].

## Methods

Analytical description of thermally induced degradation-relaxation effects was developed for spinel Cu_0.1_Ni_0.1_Co_1.6_Mn_1.2_O_4_ thick films prepared by screen-printing route as described in more detail elsewhere [17–20].

The starting bulk ceramics were synthesized by sintering technology (1040 °C, 4-h duration) using reagent grade Cu carbonate hydroxide and Ni, Co, and Mn carbonate hydroxide hydrates [21–24]. The paste was prepared by mixing powder of basic Cu_0.1_Ni_0.1_Co_1.6_Mn_1.2_O_4_ ceramics with MБ-60 glass (without Pb additives), Bi_2_O_3_ (used as inorganic binder), and some organic vehicle. The paste was printed on alumina substrates (Rubalit 708S) with a conductive Ag-Pd layer (screen printed from C1216 paste) using a manual screen-printing device equipped with a steel screen. In the final, the thick films were fired at 850 °C.

The thick-film spinel-conductor systems (prepared in such a way) were subjected to degradation testing under prolonged storage at the elevated temperature of 170 °C within a sequence of time intervals lasting from 24 to 360 h. Only 10–12 points were selected to determine the finalized relaxation kinetics for each sample. The results of aging tests were controlled by RRD, e.g., changes in electrical resistance *ΔR/R*_0_ detected under normal conditions and the confidence interval in the RRD-measuring error bar being no worse than ±0.2%. Additional deviations in some points were allowed due to faults in exact reproduction of degradation cycles in multiple sample-to-sample measurements (cooling regime from aging temperature, influence of environment and humidity, etc.). Statistical analysis testified these factors introduced additional error of about ±0.2% in *ΔR/R*_0_ values. So the overall uncertainties in electrical measurements within this aging protocol did not exceed ±0.4 to 0.5%.

With the purpose of an adequate mathematical description of thermally induced degradation-relaxation kinetics, the detected *ΔR/R*_0_ values were analyzed by *non-linear least squares curve-fitting* to the generalized decaying function [8, 9]:

2 $$ \mathrm{R}\mathrm{R}\mathrm{D} = a\left(1- \exp \left[-{\left(t/\tau \right)}^{\beta}\right]\right) $$

where *a* stands for degradation amplitude, *τ* is time constant, and *β* is *non-exponentiality index*. Numerical values of fitting parameters (*a*, *τ*, and *β*) used in this *master equation* (2) were calculated in such a way to minimize the mean-square deviations *err* (goodness of fitting) of experimental *ΔR/R*_0_ points from a model curve. As a result, under accepted uncertainties in the *ΔR/R*_0_ measurements, the final accuracy in the value of non-exponentiality index *β* was ±0.05.

The effects of conductor penetration on spinel-type ceramics were studied for fresh cuttings of the prepared thick-film structures using scanning electron microscopy (SEM) with energy-dispersive X-ray (EDX) microanalysis (LEO 982 field emission SEM), the high-quality micrographs being recorded for typical thick films with Ag or Ag-Pd alloy thick-film contacts.

## Results and Discussion

**Fig. 1:**
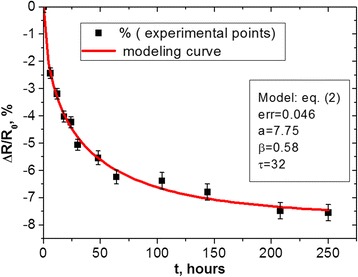
Degradation testing of RRD *ΔR/R*_0_ caused by prolonged storage at 170°C in Cu_0.1_Ni_0.1_Co_1.6_Mn_1.2_O_4_ thick films prepared with screen-printed Ag-Pd contacts (the *symbols* stand for experimental data and the *line* represents SER model curve)

**Fig. 2:**
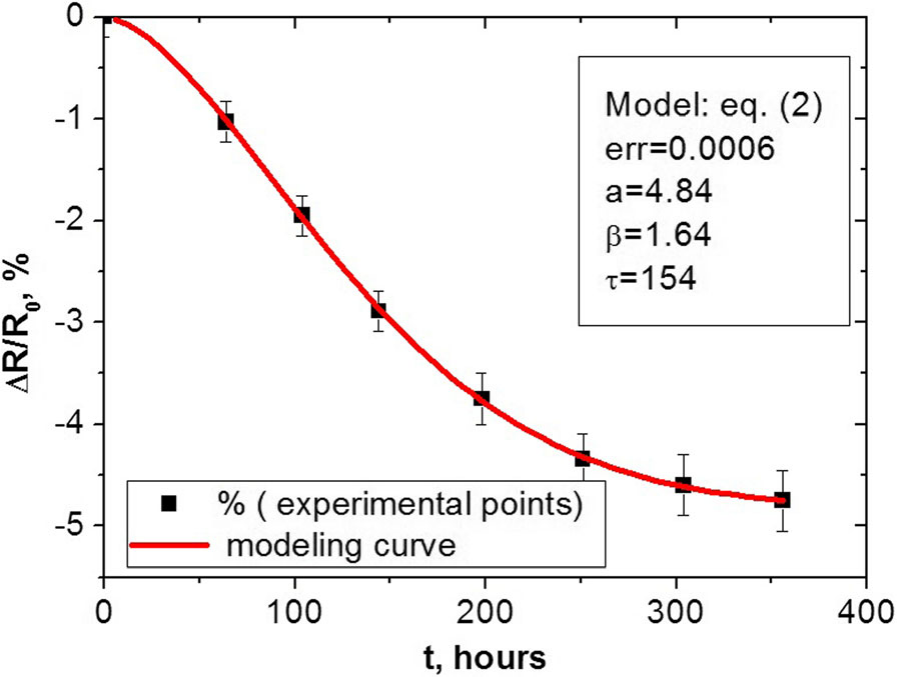
Degradation testing of RRD *ΔR/R*_0_ caused by prolonged storage at 170 °C in Cu_0.1_Ni_0.1_Co_1.6_Mn_1.2_O_4_ thick films prepared with screen-printed Ag contacts (the symbols stand for experimental data and the *line* represents CER model curve) [15, 16]

The typical curve illustrating RRD in Cu_0.1_Ni_0.1_Co_1.6_Mn_1.2_O_4_ thick films with screen-printed electrical contacts prepared of Ag-Pd alloy is shown in Fig. 1. For a comparison purpose, we also present this dependence for the same thick films with electrical contacts made of pure Ag paste (Fig. 2) plotted on the basis of previous results [15, 16].

It is seen that the degradation modeling curve for Cu_0.1_Ni_0.1_Co_1.6_Mn_1.2_O_4_ thick films with Ag-Pd contacts represents a well-known SER function (1) with a character of non-exponentiality index *β* approaching ~0.58 and effective time constant *τ* ≅ 32 h. In contrast, with change in the conductive materials of electrical contacts (so under transition to spinel ceramics-Ag thick-film system), governing degradation-relaxation kinetics drastically changed, attaining an obvious CER form with over-unity compressibility index *β* = 1.64 and time constant reaching *τ* ≅ 154 h.

**Fig. 3:**
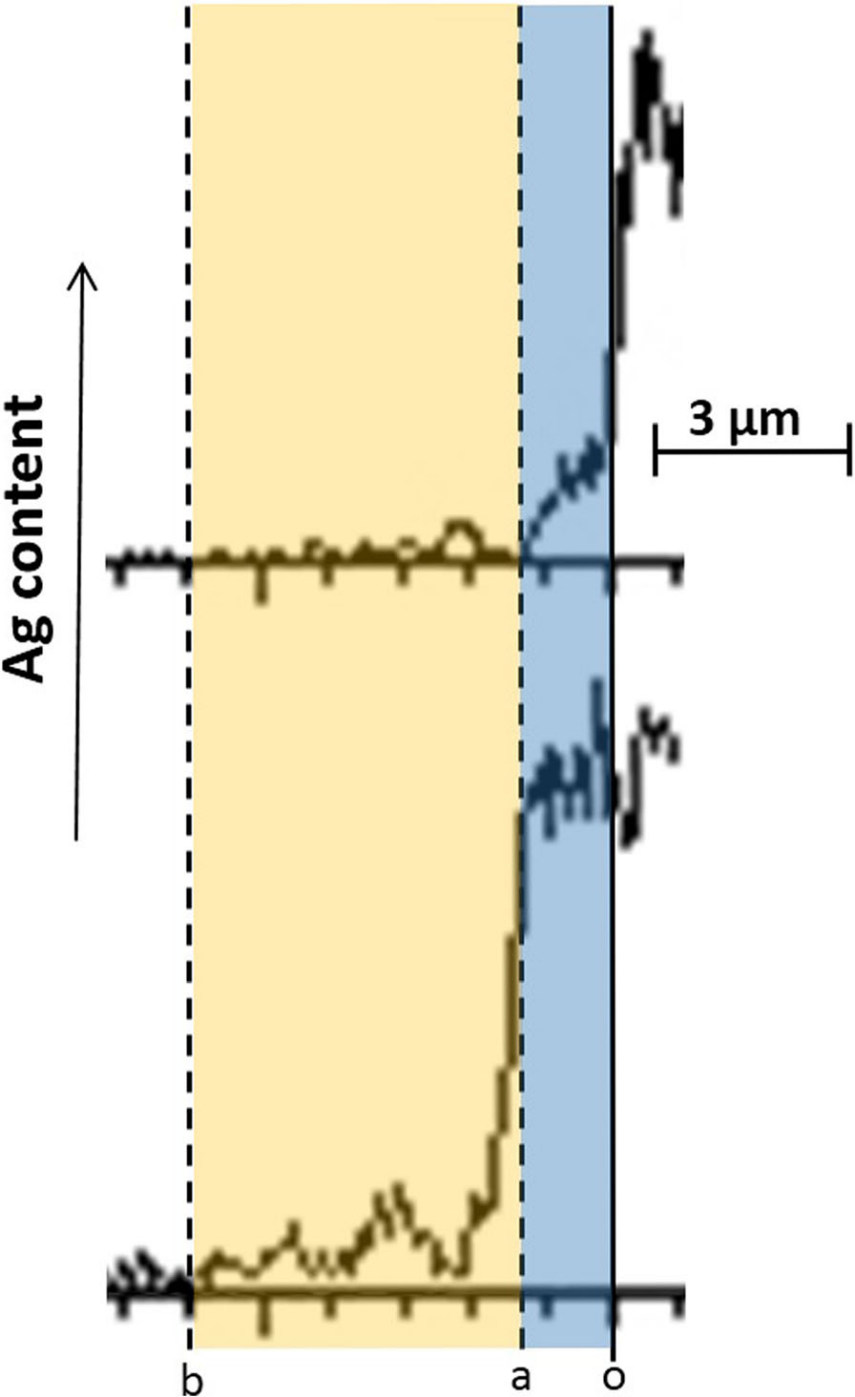
EDS profile of Ag distribution across cut section of spinel ceramics-Ag thick film (vertical *o* axis) before (*top*) and after (*bottom*) thermally induced aging tests (the *blue-distinguished oa domain* corresponds to Ag penetration in ceramics adjusted to conductor layer and the *yellow-distinguished ab domain* corresponds to further Ag penetration in deep ceramics bulk)

The EDX study of Ag distribution profiles on fresh cut sections of thick-film ceramics-Ag structures before and after degradation testing (Fig. 3) shows essential Ag penetration into ceramics. Metallic Ag appears in the regions of spinel ceramics adjusted just to conduct contact denoted by vertical *o* axis (*oa* domain in Fig. 3), as well as in a more extended deep region in ceramics bulk (*ab* domain in Fig. 3). In case of conductive Ag-Pd alloy contact, the latter domain *ab* is practically absent, corresponding to Ag penetration only into short pre-contact region *oa*.

**Fig. 4:**
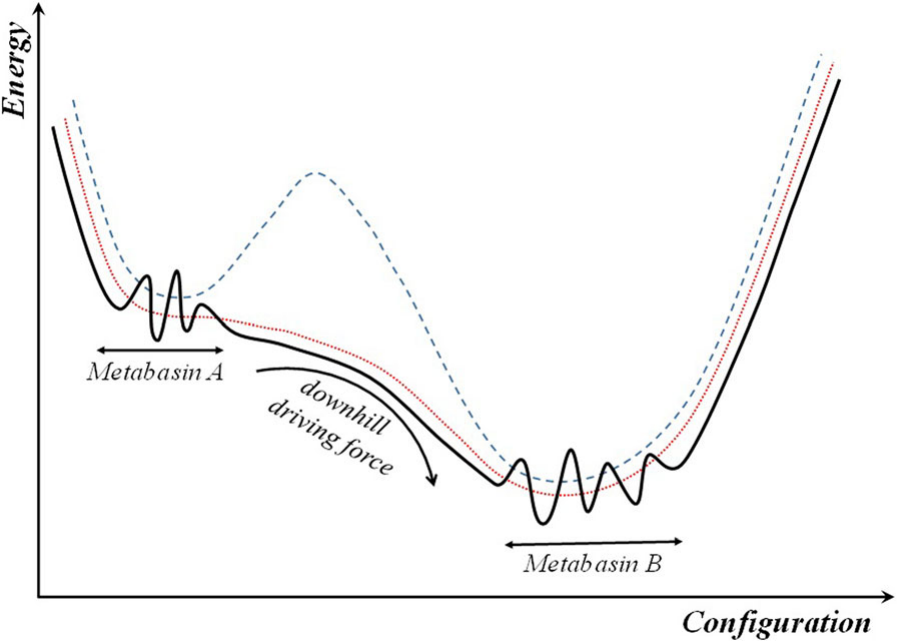
Fragment of free energy landscape of ceramics-conductor thick-film system illustrating strong downhill scenarios for relaxation due to essential Ag migration inside ceramics (non-exponential relaxation kinetics results from disappearing of inter-well barrier denoted by *dotted line* between two intermediate states of a system)

It is known that a conductive material penetrating spinel ceramics reduces electrical resistivity, thus resulting in negative RRD for such thick-film systems [15–19, 24]. This diffusive-related process is strongly thermally activated, in contrast to one’s changes in ceramics body, producing rather positive feedback in RRD due to increased defectiveness of spinel matrix [24]. The negative RRD quickly saturates with the conductive material, penetrating into ceramics as it characterizes thick films of Cu_0.1_Ni_0.1_Co_1.6_Mn_1.2_O_4_ spinel with Ag-Pd alloy contacts. Under these conditions, the whole Ag-Pd alloy behaves as one “cumulative” diffusing agent with a significantly suppressed possibility for Ag atoms migration. The resulting kinetics of such diffusive degradation-relaxation process attains strong tendency to yield SER scenarios as it is well illustrated by the fitting procedure in Fig. 1. Within the energetic landscape of a spinel ceramics-conductor system (see Fig. 4), such degradation process is revealed as subsequent transitions between a set of wells (basins) tending towards overall thermodynamic minimum within the same metabasin.

However, if Ag penetration is not sufficiently inhibited in spinel ceramics by Pd addition, as for Cu_0.1_Ni_0.1_Co_1.6_Mn_1.2_O_4_ thick films with contacts made of pure screen-printed Ag paste, the kinetics of resulting diffusive process is changed. The Ag atoms can easily migrate in spaces between crystalline ceramic grains filled with glass binder, this first stage of diffusion-related process being quickly saturated via typical SER dependence (1) with stretching exponent 0< *β* <1.

The mechanisms and physical peculiarities of Ag migration in different electronic substances have been a question for tight interests for scientists for a long time [25–31] starting with the pioneering work of Kohman et al. [25]. In a most generalized opinion, this process is attributed to continuous generation and accumulation of Ag ions, their recharging into metallic silver followed by formation of cloud-like layers or dendritic migration outgrowths [29–31]. Whichever the case, by penetrating intergranual spaces in a thick-film body, the Ag ions attain a possibility for further migration between (and probably into) bulk grains composed of Cu_0.1_Ni_0.1_Co_1.6_Mn_1.2_O_4_ spinel, thus leading to the second stage diffusion.

In such a way, the ceramics material evolving crystalline Cu_0.1_Ni_0.1_Co_1.6_Mn_1.2_O_4_ grains, intergranual barriers, and structurally intrinsic pores forms a specific *jammed system*, where strong *downhill scenarios* appear as an input from additional silver penetration deep in ceramics bulk. Therefore, the overall diffusion-limited relaxation process in the studied thick-film system occurs as a *two-step penetration* of a conductive agent (Ag) into spinel ceramics. Within the free energy landscape of the spinel ceramics-conductor system shown in Fig. 4, this process can be conditionally reflected as an obvious decreasing tendency between two groups of basins (A and B metabasins). The similar processes are proper to protein chains affected by folding [10–14] or temperature-induced volume phase transitions in poly(*N*-isopropylacrylamide) [32]. As a result, the overall kinetics of thermally induced degradation-relaxation transformations tends towards CER behavior with over-unity compressibility index *β* as it is well illustrated in Fig. 2.

In general, the penetration of a metallic conductor inside the solid matrix can be illustrated as a transition between two free energy wells corresponding to different metastable states of a system separated by a high enough barrier. In this case, the governing kinetics is known to be a single-exponential one [14]. The non-exponential relaxation implies disappearing of inter-well barrier and subsequent flattening of rugged free energy surface of a system as it is well depicted in Fig. 4. In a non-equilibrium state under weak perturbation in a system, the corresponding kinetics will be SER [14], as it characterizes inhibited Ag migration in ceramics-conductor films with Ag-Pd alloy contacts. In case of a more pronounced structural perturbation in a thick-film system under essential conductive phase migration, as for thick films with Ag contacts, the resulting kinetics is CER. From a purely physical point, this means that a strong *downhill driving force* appears between metastable states on a free energy diagram (A and B metabasins in Fig. 4) composed of a sequence of flattened but still rugged distinct basins.

In terms of heterogeneity [11], the non-exponential relaxation kinetics can be described by heterogeneity factor *h* (which is inverse stretching factor 1/*β*), giving the degree of system deviation from homogeneous single-exponential relaxation function. This parameter for SER kinetics in Fig. 1 is significantly greater than 1 (*h* = 1.72), reflecting a complex glassy process dominated by multiple local minima. For CER kinetics in Fig. 2, the heterogeneity factor *h =* 0.61 corresponds to non-glassy (strongly non-exponential) kinetics in Ag-penetrating Cu_0.1_Ni_0.1_Co_1.6_Mn_1.2_O_4_ ceramics.

In fact, *stretched-to-compressed exponential kinetics crossover* appears in the studied thick-film ceramics-conductor system due to structural perturbation caused by diffusing (Ag atoms) agents. In case of photo-switchable α-helix [14], the change in the folding kinetics from SER (with *β* = ~0.7) to CER (with *β* = ~1.3) was explained by protocol of experimental measurements performed with respect to most sensitive employed observables (so the stronger downhill force results in larger stretching factor *β* and correspondingly more compressed relaxation kinetics). In polymer nanocomposites such as Au [6] or Al_2_O_3_ [7] nanoparticles embedded in polymethylmethacrylate, the CER kinetics with non-exponentiality index *β* close to 1.4–1.9 (in dependence on nanoparticle content) appears at lowering the temperature below glass transition. It is noteworthy that the same order of *β* (which serves as a measure of internal stress) defined by *stretched-to-compressed exponential kinetics crossover* for our thermally relaxing thick-film-Ag system, photo-switchable folded α-helix [14], or polymer nanocomposites [6, 7] testifies in a favor of close similarity in the responsible phenomenological models.

In many disordered systems prepared in an out-of-equilibrium state by quenching from a liquid, the internal stresses are inevitably built in at the jamming transition [1, 2]. These stresses relaxing at further experimental conditions serve as a source for unusual (or “strange”) CER, which is non-diffusive in its microstructure origin. In case of our thick films (e.g., composites formed of spinel ceramics contacting with Ag or Ag-Pd conductor), this type of relaxation (CER) dominates under a condition of dynamic inhomogeneities caused by Ag atoms penetration deep in ceramics bulk. This undoubtedly diffusive process (which typically is described in disordered solids as SER) is only the initiating stage of the overall relaxation tending a system towards equilibrium, while the final ultraslow stage attains faster than the exponential CER form. The relaxation of internal stresses has no direct relation to this first stage diffusive motion, thus being principally different in its physical origin. Thus, the driving microstructure mechanisms responsible for CER are known to be related to stresses collapse due to soft contacts between spheres (as in case of concentrated emulsions and lamellar gels), local topological rearrangements (as in foams), elastic relaxation of topological defects like dislocations or grain boundaries (as for micellar crystals), etc. [1]. Typically, such structural rearrangements occur on length scales of over-micron distances [1]. Thus, it should be admitted reasonably that Ag atoms penetrating spinel Cu_0.1_Ni_0.1_Co_1.6_Mn_1.2_O_4_ ceramic body, mainly in a vicinity of intergranual boundaries, create specific micron-sized bridges between ceramics grains, thus increasing the electrical conductivity of a whole thick-film system. More detailed microstructure nature of these Ag-based structural entities is still under question.

## Conclusions

Structural inhomogeneities due to metallic diffusing agents (conductive Ag and Ag-Pd alloys) embedded in Cu_0.1_Ni_0.1_Co_1.6_Mn_1.2_O_4_ spinel thick films are defined as decisive factors governing non-exponential relaxation kinetics in these nanocomposites revealed in negative relative resistance drift under thermally induced testing at 170 °C. Principally, different types of degradation-relaxation kinetics are detected for the same Cu_0.1_Ni_0.1_Co_1.6_Mn_1.2_O_4_ spinel thick-film ceramics in dependence on contacting metallic conductors. If Ag migration is significantly inhibited in spinel ceramics by Pd addition due to conductive Ag-Pd alloy, the governing kinetics attains a stretched-exponential behavior with stretching exponent *β* = ~0.6, which is typical for one-stage diffusion in structurally dispersive media. Under essential Ag penetration into ceramics, as for thick-film systems with Ag contacts, the degradation-relaxation kinetics drastically changed attaining an obvious compressed exponential form with over-unity compressibility index *β* = 1.64. The resulting kinetics in this case is thought to be attributed to a two-step diffusing process originating from Ag penetration deep into spinel ceramics.

## Abbreviations

CER: Compressed exponential relaxation
EDX: Energy-dispersive X-ray
RRD: Relative resistance drift
SEM: Scanning electron microscopy
SER: Stretched-exponential relaxation
